# *Halodule uninervis* extract facilitates the green synthesis of gold nanoparticles with anticancer activity

**DOI:** 10.1038/s41598-024-81875-0

**Published:** 2025-02-04

**Authors:** Nadine Wehbe, Joelle Edward Mesmar, Riham El Kurdi, Ali Al-Sawalmih, Adnan Badran, Digambara Patra, Elias Baydoun

**Affiliations:** 1 Department of Biology, American University of Beirut, Beirut, Lebanon; 2 Department of Chemistry, American University of Beirut, Beirut, Lebanon; 3 Marine Sciences Station, University of Jordan, Aqaba, Jordan; 4 Department of Nutrition, University of Petra, Amman, Jordan

**Keywords:** Inorganic chemistry, Physical chemistry

## Abstract

Gold nanoparticles (AuNPs) have been utilized in a plethora of applications due to their unique optical properties, high stability, and great biocompatibility. The use of plant extracts in the biosynthesis of AuNPs has emerged as an eco-friendly, inexpensive, and nontoxic approach. In the present study, the ethanolic extract of *Halodule uninervis* (HUE) was used for the first time in the green synthesis of AuNPs. The biogenic AuNPs were characterized using several spectroscopic and microscopic techniques and their anticancer potential was investigated. They displayed a surface plasmon resonance (SPR) band at 550 nm. Scanning electron microscopy (SEM) and Dynamic light scattering (DLS) analysis proved the synthesis of small and spherical AuNPs with a zeta potential of − 27.66 mV. X-ray diffraction (XRD) pattern established the crystalline nature of AuNPs. Thermogravimetric analysis (TGA) and Fourier transform infra-red spectroscopy (FTIR) data confirmed the role of HUE in the capping and stabilization of the manufactured AuNPs. The produced AuNPs exhibited anticancer activity against several human cancer cell lines, highlighting their potential efficacy as anticancer therapeutic agents. Further analysis revealed that this observed cytotoxicity is mediated via the induction of apoptosis. Overall, our data validate the effective use of *H. uninervis* extract in the biosynthesis of AuNPs with significant biological properties.

## Introduction

Nanotechnology, which deals with creating novel nanomaterials, has emerged as a promising tool with applications in various fields. Indeed, metallic nanoparticles have been particularly exploited in the food industry, agriculture sector, cosmetology, textile manufacturing, as well as the biomedical field^1^. And gold nanoparticles (AuNPs) have been receiving much attention due to their unique physiochemical properties, such as localized surface plasmon resonance (LSPR), chemical inertness, high stability, and great biocompatibility. Accordingly, AuNPs possess great potentials in biosensing^2^, tissue imaging^3^, photothermal therapy^4^, antimicrobial application^5^, targeted drug delivery^6^, and cancer detection and treatment^7^.

Conventional synthesis of AuNPs involves chemical processes through reduction reactions^8,9^, or physical routes such as evaporation-condensation and laser ablation^10,11^. The use of these methods is often associated with a number of challenges, including elevated cost, high time and energy consumption, intense labor, and risk of toxicity. This mandated the development of new approaches for the safe production of AuNPs. In recent years, green synthesis routes have been employed to prepare nanoparticles that are eco-friendly, inexpensive, nontoxic, and with enhanced activities. In fact, various biomaterials such as algae, bacteria, fungi, and plant extracts are used as reducing agents in the green synthesis of nanoparticles^12^. Moreover, plant extracts grant numerous benefits over other biological approaches due to their widespread accessibility, low maintenance needs, biosafety, and cost-effectiveness. *Curcuma pseudomontana*^13^, *Hibiscus rosa-sinensis*^14^, *Nigella arvensis*^15^, and *Ziziphus nummularia*^16^ are some examples of plants that have been successfully utilized in the green synthesis of AuNPs.

Besides terrestrial plants, the marine environment is gaining increased attention in the field of nanotechnology. Seagrasses, also known as marine angiosperms, are the only flowering plants to recolonize the seabed. They play a role in providing a habitat and breeding ground for many marine organisms, supporting food security, and stabilizing coastal sediments^17^. In addition to their significant role in the marine ecosystem, seagrasses have been used in folk medicine for the treatment of fever, muscle pains, skin diseases, and wounds^18^. *Halodule uninervis* of the family *Cymodoceaceae* is an abundant seagrass species localized in a wide range of habitats that can withstand stressful conditions owing to the presence of diverse secondary metabolites. These metabolites, including flavonoids, phenols, and tannins, provide the plant with various pharmacological properties. *Halodule uninervis* extracts have been reported to have antidiabetic^19,20^, antimicrobial^21^, antioxidant^22,23^, and anticancer^24^ properties.

The present study aims to utilize *Halodule uninervis* ethanolic extract (HUE) for the green synthesis of AuNPs and to test their anticancer potential. We report the successful synthesis of AuNPs using HUE as a reducing and capping agent. Our synthesized AuNPs exhibit high anticancer potential by inhibiting the proliferation of several cancer cell lines in vitro through the possible induction of apoptosis.

## Materials and methods

### Preparation of *Halodule uninervis* ethanolic extract (HUE)

*Halodule uninervis* was obtained from the Gulf of Aqaba in Jordan. The plant was identified by Mohammad Al Zein, a plant taxonomist at the Biology Department, American University of Beirut (AUB). A voucher specimen under number JO 2023-02 has been deposited at the Post Herbarium, AUB. The leaves were thoroughly washed, air-dried in the dark, and ground into fine powder. Powdered leaves were suspended in 80% ethanol for 72 h in the dark with continuous shaking. Finally, the solution was filtered, dried using a rotary vacuum evaporator, and lyophilized. The obtained powder was used for the synthesis of gold nanoparticles.

### Green synthesis of gold nanoparticles using HUE

For the green synthesis of AuNPs, HUE and gold (III) chloride trihydrate (HAuCl_4_.3H_2_O) (Acros Organic) were mixed at a ratio of 2:1 in 20 mL double distilled water at 70–80 °C. The solution was sonicated for 30 min until its color changed from yellow to dark purple. Then, the solution was centrifuged at 15,000 rpm for 15 min. The collected AuNPs were dissolved in double distilled water and lyophilized, and the obtained powder was stored at 4 °C for further use.

### Characterization of gold nanoparticles

The optical absorption of the synthesized AuNPs was measured using a UV–Visible spectroscopy (JASCO V-570 UV–Vis–NIR spectrophotometer) in the 450–800 nm wavelength range at room temperature.

The shape of AuNPs was determined using a scanning electron microscopy (SEM) with a MIRA3 LMU coupled to OXFORD EDX detector. Briefly, a drop of diluted AuNPs was applied onto a carbon-coated aluminum stub, air-dried, and observed with SEM.

The size distribution and zeta potential of AuNPs solution was assessed using the dynamic light scattering (DLS) (Brookhaven Instruments Corps) technique with a laser source operating at 658 nm and a PMT detector (HAMAMATSU, HC120-30). The dust was adjusted to 40 and the program used was 90Plus Particle Sizing Software Ver. 5.23.

The thermal stability of HUE and AuNPs was assessed by thermogravimetric analysis (TGA) using a Netzsch TGA 209 under a Nitrogen atmosphere in a temperature range from 30 to 900 °C with 15 K/minute step size. This measurement was done on 5 mg of the samples in Aluminum oxide (Al_2_O_3_) crucibles.

The crystallographic structure of AuNPs was examined by X-ray diffraction (XRD) pattern using a D8 advance X-ray diffractometer by Bruker. Samples were recuperated as fine powder and placed on the zero-background holder. The scan type was coupled 2θ/θ for 2θ between 30° and 80°, with increments of 0.02°.

The nature of nanoparticle-associated molecules was investigated by Fourier-transform infrared attenuated toral reflectance (FTIR-ATR) measurements. FTIR-ATR spectra were obtained using a Bruker Tensor 27 FT-IR equipped with a diamond lens ATR module.

### Cell culture

Human breast cancer cells MDA-MB-231 (American Tissue Culture Collection, ATCC, Manassas, VA) and Capan-2 human pancreatic cancer cells (Cell Line Service, CLS, Eppenlheim, Germany) were maintained in DMEM high-glucose medium supplemented with 10% fetal bovine serum (FBS) (both from Sigma-Aldrich, St. Louis, MO, USA) and 1% penicillin/streptomycin (Lonza, Switzerland).

Human colorectal cancer cells HCT116 (ATCC, Manassas, VA) and 22RV1 human prostate cancer cells (ATCC, Manassas, VA) were maintained in RPMI-1640 medium (Sigma-Aldrich, St. Louis, MO, USA) supplemented with 10% fetal bovine serum (FBS), 1% penicillin/streptomycin, and 1% sodium pyruvate.

All cells were cultured in a humidified incubator at 37 °C and 5% CO_2_.

### MTT cell viability assay

Cells were seeded in 96-well plates (5 × 10^3^ cells per well) and incubated for 24 h until they were 30–40% confluent. Cells were then treated with 0, 10, 25, 50, and 75 µg/mL of AuNPs and incubated for 24, 48, and 72 h. Cell viability was measured by 3-(4,5- dimethylthiazol-2-yl)-2,5-diphenyltetrazolium bromide (MTT; Sigma-Aldrich, St. Louis, MO, USA) reduction assay.

MTT assay is commonly used in the assessment of cell viability and proliferation. In this context, the yellow MTT reagent is reduced by “healthy” cells to purple formazan crystals active metabolic processes in the mitochondria. During apoptosis, the cells undergo several morphological and biochemical changes, which include the loss of mitochondrial membrane integrity leading to a decrease in metabolic activity, and consequently a decrease in the formation of formazan. Cell growth was determined as the proportional viability of treated cells in comparison with vehicle (DMSO)-treated ones, where the viability was presumed to be 100%. The assay was performed in triplicate and repeated three times. Data are presented as mean values ± SEM.

### Microscopic analysis of apoptotic morphological changes

MDA-MB-231 cells were grown in 6-well plates in the presence or absence of the indicated concentrations of HUE. Morphological characteristics of apoptotic cells were observed after 24 h using an inverted phase-contrast microscope at ×10, ×20, and ×40 magnifications.

4′, 6-diamidino-2-phenylindole, dihydrochloride (DAPI) (Cell signaling #4083) staining was used to determine changes in nuclear morphology. Cells were grown in 12-well plates in the presence or absence of the indicated concentrations of HUE for 24 h. Cells were then fixed with 4% formaldehyde, stained with DAPI, and visualized by fluorescence microscopy.

### Statistical analysis

Data were statistically evaluated using two-way ANOVA (with Tukey–Kramer’s post hoc test). Data are presented as mean ± SEM. A p-value of less than 0.05 was considered as statistically significant.

## Results and discussion

### Green synthesis of gold nanoparticles using HUE

**Fig. 1:**
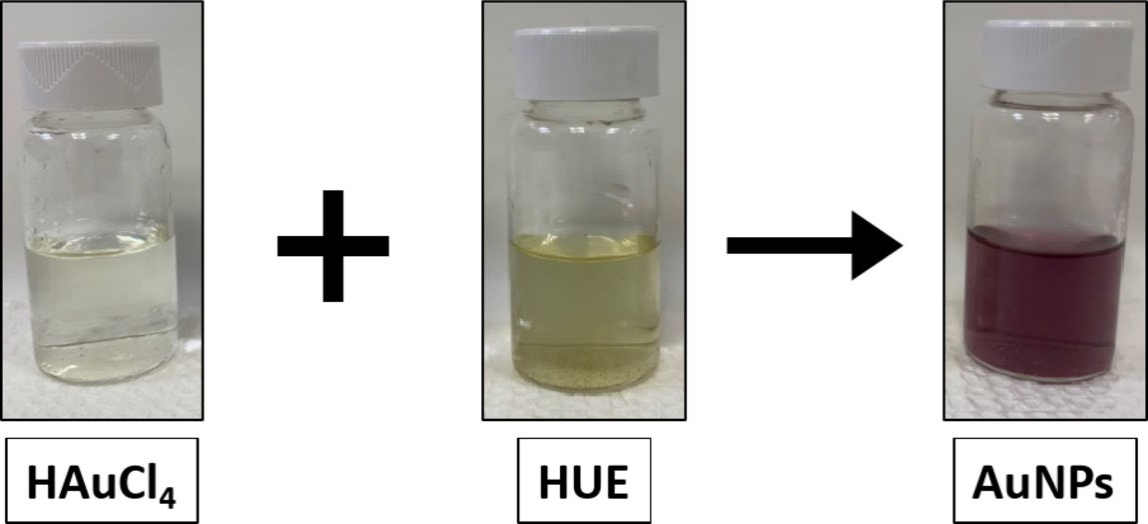
Color change from yellow (Au^3+^) to purple (Au^0^) indicates the formation of AuNPs in the presence of *Halodule uninervis* extract.

The green synthesis of AuNPs was achieved using HUE plant extract as a reducing agent. HUE promotes the reduction of aqueous gold ions (Au^3+^) into gold nanoparticles (Au^0^ precipitate). The change in the color of the solution from yellow to dark purple indicates the successful formation of biogenic AuNPs (Fig. 1). We susupect that the hydroxyl, carbonyl and acid groups present various phytochemical is generally responsible of the reduction reaction.

**Fig. 2:**
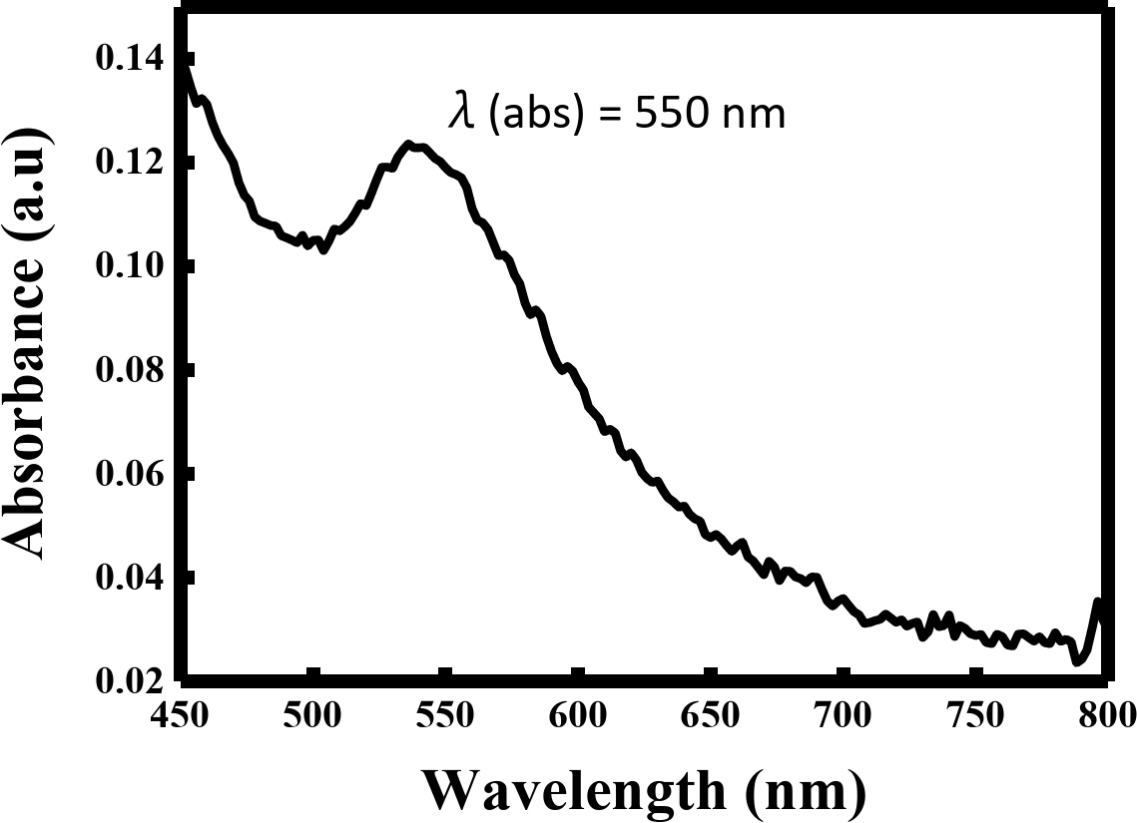
UV-visible spectrum of *Halodule uninervis* synthesized AuNPs.

The synthesis of AuNPs was further confirmed by measuring the localized surface plasmon resonance (LSPR) using UV–Visible spectroscopy. LSPR is a distinct optical phenomenon of AuNPs that refers to the collective oscillations of free electrons confined to the surface of plasmonic nanoparticles when excited with an incident light at a specific wavelength^25,26^. This feature of plasmonic nanoparticles facilitates their application in biosensing, cellular imaging, and immunoassays^27^. LSPR of AuNPs appears as a sharp absorbance band in the visible region wavelength of 500–600 nm^28^. As shown in Fig. 2, the prepared AuNPs absorbed at λ_abs_ = 550 nm, verifying the successful preparation of AuNPs. The absorption peak and intensity of LSPR spectrum depends on the size and shape of synthesized AuNPs^29,30^. A red-shift in the absorbance peak corresponds to the formation of AuNPs with large diameter and uneven shape. The absorbance peak of our synthesized AuNPs at λ_abs_ = 550 nm (Fig. 2) insinuates the formation of spherical nanoparticles with a small diameter.

### Characterization and spectroscopic analysis of the biosynthesized gold nanoparticles

#### Morphology and size distribution analysis

**Fig. 3:**
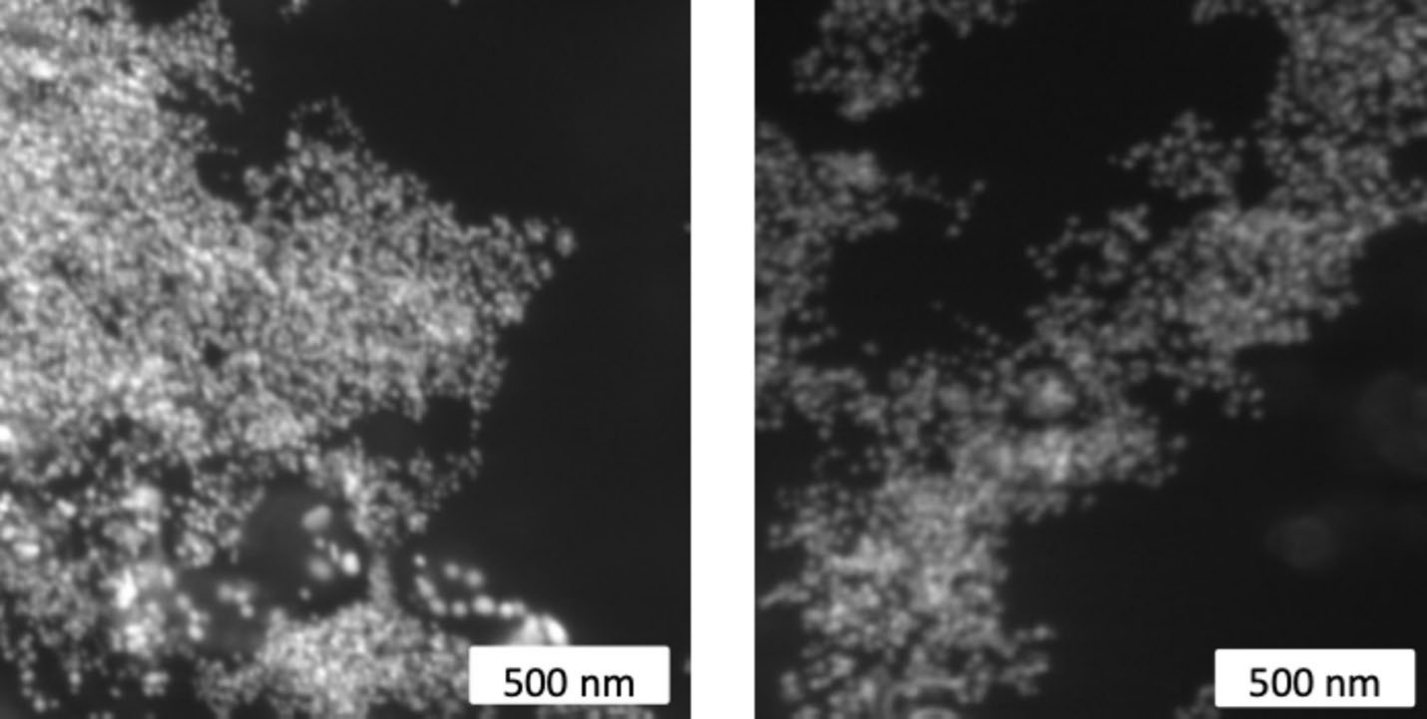
Scanning electron microscopy (SEM) micrograph showing the morphology of *Halodule uninervis* synthesized AuNPs at 500 nm scale resolution.

UV–Visible spectroscopy data revealed the formation of small AuNPs with a spherical shape. The morphology and size of our biosynthesized AuNPs were further confirmed by scanning electron microscopy (SEM) and dynamic light scattering (DLS), respectively. SEM confirmed the formation of spherical AuNPs, which are uniform and less than 50 nm in size (Fig. 3). Images also showed some degree of aggregation between AuNPs.

**Fig. 4:**
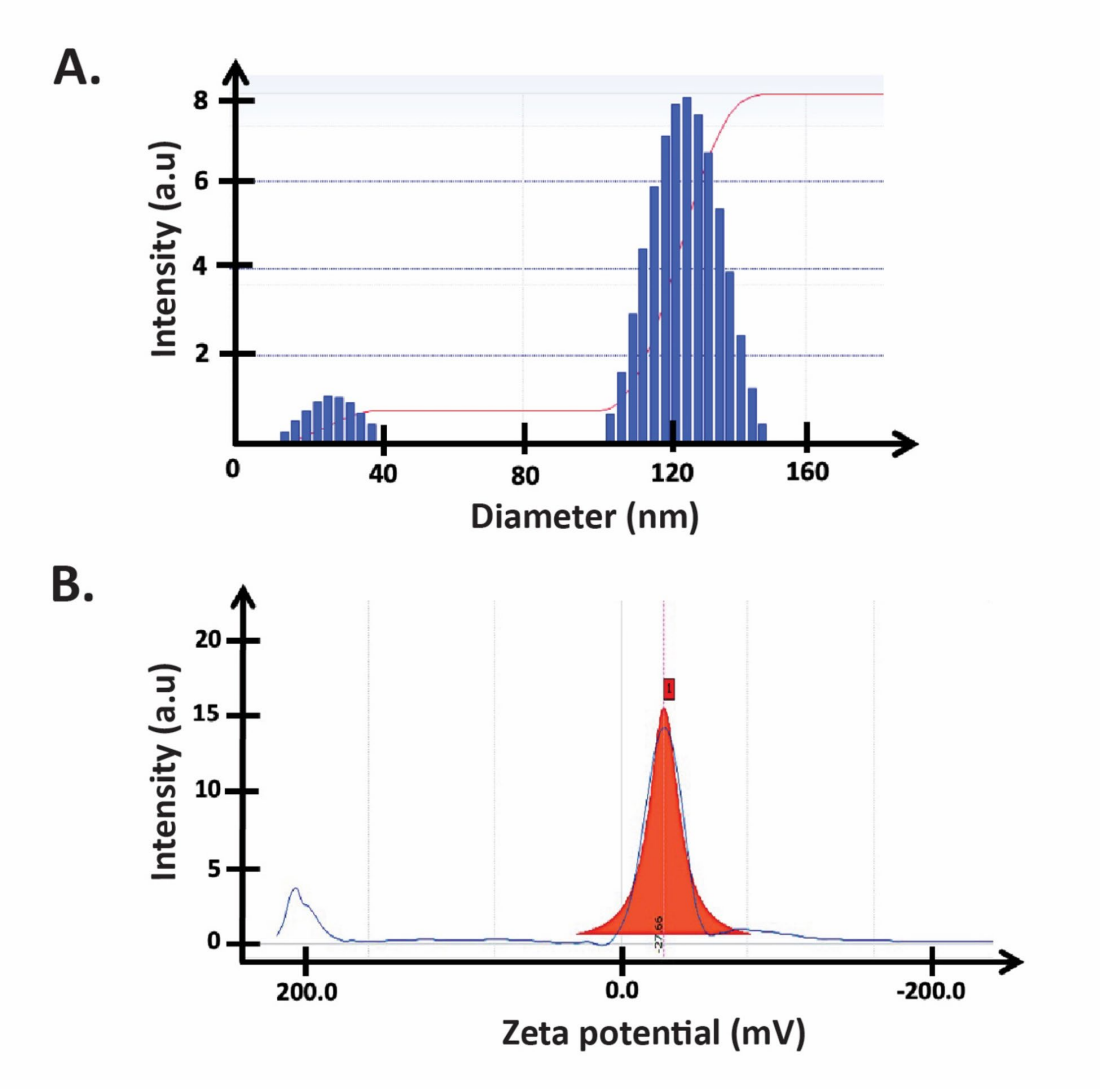
Dynamic light scattering (DLS) of *Halodule uninervis* synthesized AuNPs. (**A**) Size distribution and (**B**) Zeta potential.

Dynamic light scattering (DLS) was conducted to confirm the small size of AuNPs, their size distribution, and aggregation state. Indeed, as shown in Fig. 4A, the size of our biosynthesized AuNPs was between 10 and 50 nm. The presence of sizes between 200 and 300 nm is attributed to the size of AuNPs aggregates as indicated in SEM images. The polydispersity index (PDI) was 0.182, indicating the formation of uniform AuNPs.

A Zeta potential measurement of − 27.66 mV indicates that the produced nanoparticles exhibit colloidal dispersion and are stable in suspension (Fig. 4B).

#### Crystallinity analysis

**Fig. 5:**
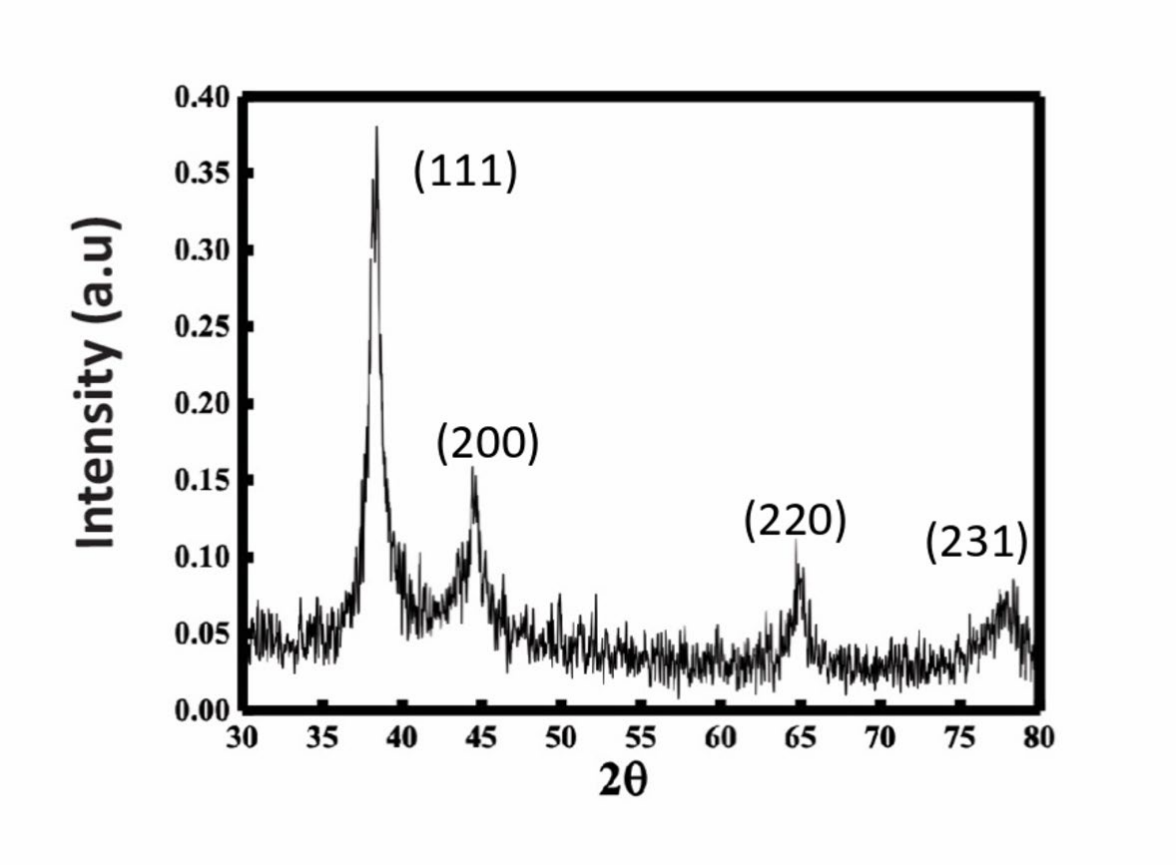
X-ray diffraction (XRD) of *Halodule uninervis* synthesized AuNPs.

X-ray diffraction (XRD) technique was used to establish the crystallographic structure of AuNPs. As shown in Fig. 5, the obtained diffractograms revealed four distinct characteristic peaks at 38.5°, 44.5°, 64.6°, and 77.5°. All peaks correspond to the (111), (200), (220), and (311) Bragg reflections of the face-centered cubic (FCC) structure, which is a typical pattern for AuNPs and consistent with previous studies of biosynthesized AuNPs using different extracts^31–33^. The observed diffraction peaks are identical to those reported for standard gold metal according to the International Centre for Diffraction Data (ICDD, reference code 00-004-0784)^34,35^. Furthermore, the absence of other peaks indicates the high purity of AuNPs. Thus, the XRD pattern provides strong evidence that the produced AuNPs were of pure crystalline gold.

#### Stability and thermal behavior

**Fig. 6:**
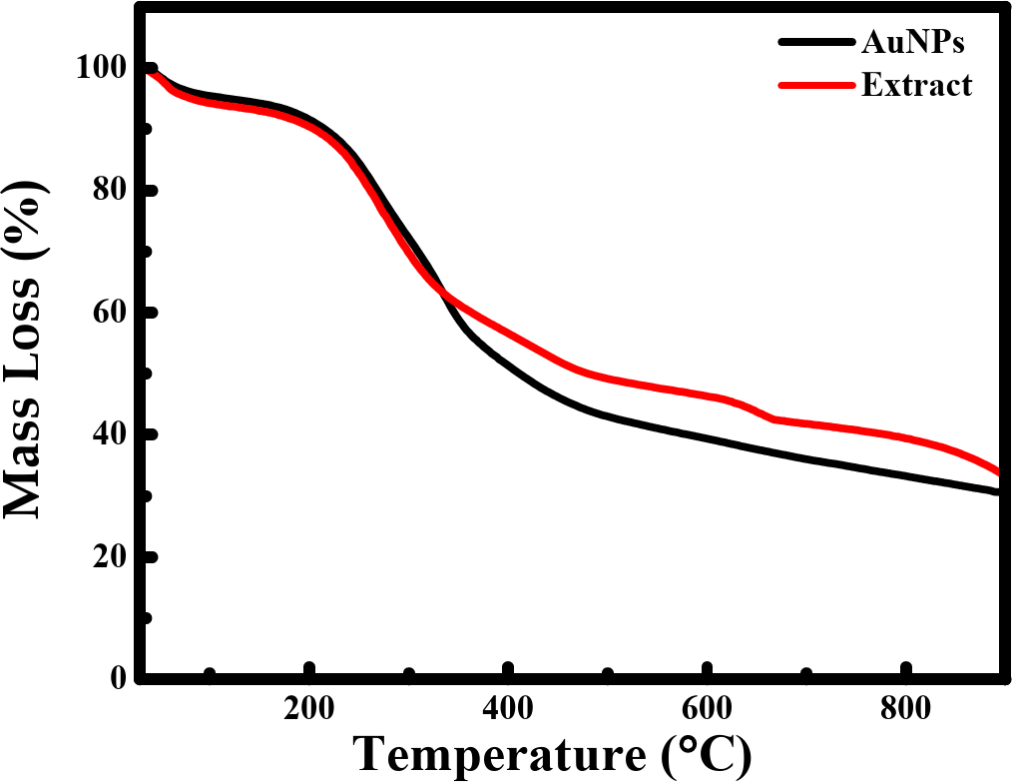
Thermogravimetric analysis (TGA) of *Halodule uninervis* synthesized AuNPs.

The thermogravimetric analysis (TGA) graphs of HUE extract and AuNPs showed a steady weight loss in the temperature range of 150–600 °C (Fig. 6). The weight loss between 100 and 200 °C refers to the loss water. Furthermore, the similar weight loss behavior pattern of AuNPs and HUE suggests that the 60% weight loss between 200 and 600 °C could be attributed to the degradation of the HUE extract. This indicates that, in addition to its role as a reducing agent, our extract has a protective role on the surface of AuNPs by serving as a stabilizing/capping agent.

#### Molecular capping analysis

**Fig. 7:**
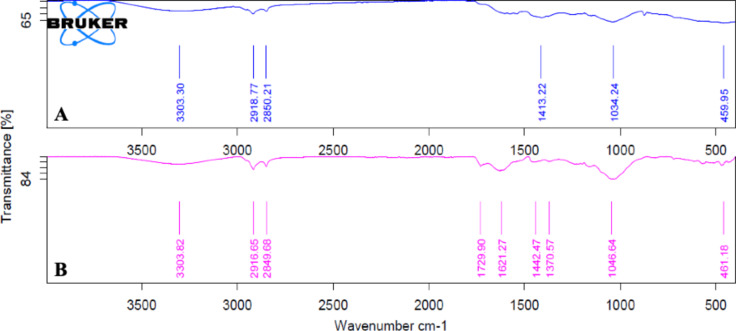
Fourier transform infrared (FTIR) spectra of (**A**) *Halodule uninervis* extract and (**B**) the biosynthesized AuNPs.

Fourier Transform Infrared Spectroscopy (FTIR) spectral measurements were performed in the range of 400–4000 cm^-1^ to identify the possible functional groups of the extract involved in the reduction, capping, and stabilization of AuNPs. Figure 7 shows an extensive resemblance between the FTIR spectra of HUE extract and AuNPs, indicating the presence of similar functional groups, which could be key players in the biosynthesis of AuNPs. For instance, the broad band at 3303 cm^-1^ is usually associated with phenolic hydroxyl groups from tannins and flavonoids^1,7^. Moreover, small peaks ranging from 1600 to 500 cm^-1^ are characteristics of polyphenols^1^. Overall, the presence of these hydroxyl and carbonyl containing compounds could explain the role of secondary metabolites found in HUE extract in the reduction, capping, and stabilization of AuNPs^4^. A summary of the bands’ positions and their possible corresponding functional groups is illustrated in Table 1.

**Table 1:** Identification of the main peaks found in the FTIR spectra of the *Halodule uninervis* extract, HAuCl_4_, and *Halodule uninervis synthesized AuNPs.*

Peak	Bands (cm^− 1^)		Possible functional groups	References
	Extract	AuNPs		
1	3303.30	3303.82	O–H	(31, 32, 51, 54)
2	2918.77; 2850.21	2916.65; 2849.68	C–H or C–H_2_	(54–56)
3		1729.90	C = O	(31, 55, 57, 58)
4		1621.27	COO–	(50, 55, 59)
5	1413.22	1442.47	C–H or C–H_2_	(31, 57)
6		1370.57	CH_3_	(50, 56)
7	1034.24	1046.64	C–O	(31, 51, 58, 59)

### Anticancer activity of the biosynthesized gold nanoparticles

**Fig. 8:**
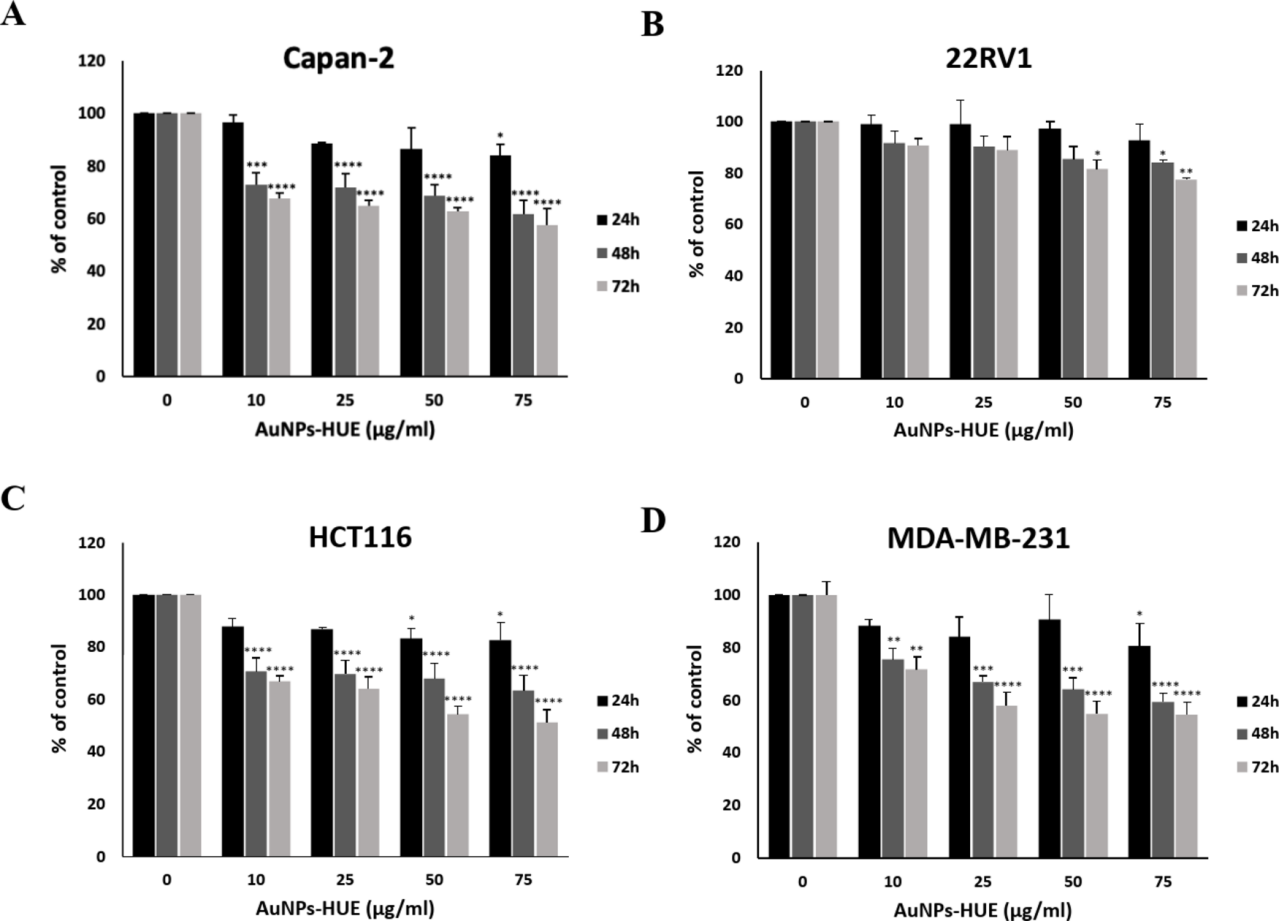
*Halodule uninervis* synthesized AuNPs inhibit cellular proliferation of (**A**) Capan-2 pancreatic, (**B**) 22RV1 prostate, (**C**) HCT116 colorectal and (**D**) MDA-MB-231 breast cancer cells. Cells were treated with and without the indicated concentrations of AuNPs for 24, 48, and 72 h. DMSO was used as the vehicle control. Viability was examined using the metabolic-dye-based MTT assay. Data represent the mean ± SEM of three independent experiments (*n* = 3) and are expressed as a percentage of the corresponding control cells. (*p < 0.05, **p < 0.005, ***p < 0.001, ****p < 0.0001).

After the successful synthesis and characterization of AuNPs using *Halodule uninervis* ethanolic extract, we evaluated their anticancer potential against four human cancer cell lines: human pancreatic cancer cell line (Capan-2), human prostate cancer cell line (22RV), human colorectal cancer cell line (HCT116), and human breast cancer cell line (MDA-MB-231). The effect of different concentrations (0, 10, 25, 50 and 75 µg/mL) of AuNPs on the viability of cancer cells was assessed at 24, 48 and 72 h of treatment. Results showed that AuNPs decreased cell viability in a concentration- and time-dependent manner, with the exception of 22RV1 prostate cancer cells (Fig. 8). For instance, at 48 h of treatment, cell viability using 10, 25, 50 and 75 µg/mL AuNPs against MDA-MB-231 cells was 75.31 ± 4.2, 66.76 ± 2.3, 63.89 ± 4.5, and 59.03 ± 3.7 that of control cells, respectively (Fig. 8D). The half-maximal inhibitory concentration (IC_50_) values for all cell lines are listed in Table 2. Our results are in accordance with previous research, where seagrasses have been used to synthesize metallic nanoparticles. Those biosynthesized nanoparticles also exhibited anticancer activities against several human cancer cell lines^36–38^. For instance, silver nanoparticles (AgNPs) synthesized using the marine plant *Cymodocea serrulata* exhibited a potential anticancer activity against human breast cancer MCF-7 and human cervical cancer HeLa cell lines, which was characterized by a concentration-dependent inhibition of cell viability^36,38^. *C. serrulata* was also used for the biosynthesis of zinc oxide nanoparticles (ZnONPs), which were found toxic against human lung cancer A549 and H520 cell lines^37^. It is worth noting that many plant extracts have been also used for the green synthesis of silver nanopartilces (AgNPs) with potent anticancer as well as antimicrobial potentials^39–43^. Targeting cancer cells with a combination of biogenic AuNPs and AgNPs could thus ehance their therapeutic effect due to their complementary properties.

**Table 2:** The half-maximal inhibitory concentration (IC_50_) values for all cell lines after 72 h of treatment with *Halodule uninervis* synthesized AuNPs.

Capan-2	22RV1	HCT116	MDA-MB-231
144.4 µg/mL	262.03 µg/mL	89.97 µg/mL	92.59 µg/mL

**Fig. 9:**
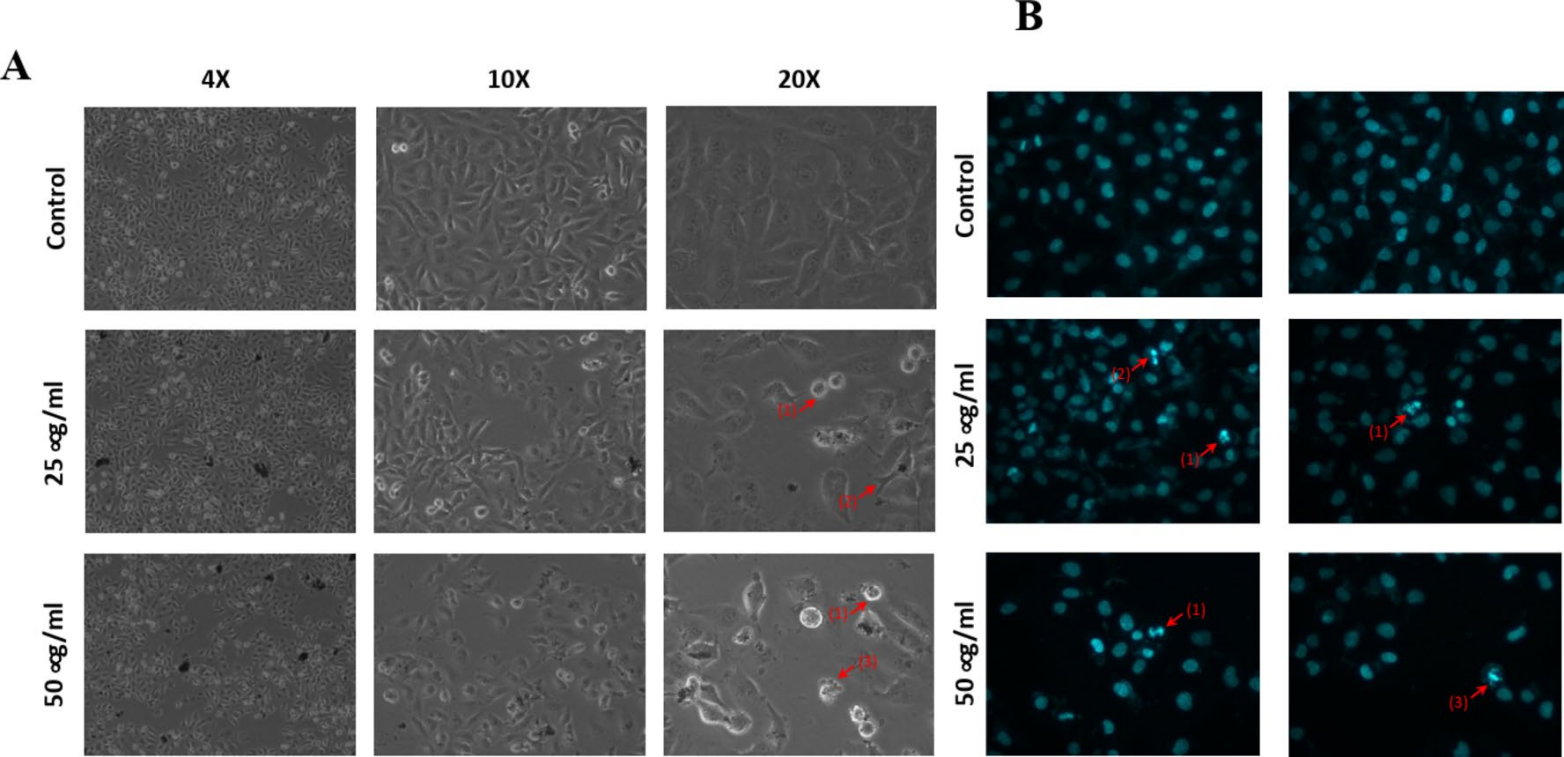
*Halodule uninervis* synthesized AuNPs induce apoptosis in MDA-MB-231 cells. (**A**) MDA-MB-231 cells were treated with or without the indicated concentrations of AuNPs for 24 h. Morphological changes were observed by light microscopy. Arrows show (1) membrane blebbing, (2) echinoid spikes, and (3) apoptotic bodies. (**B**) MDA-MB-231 cells were incubated with or without the indicated concentrations of HUE for 24 h and stained with 4ʹ, 6-diamino-2-phenylindole (DAPI) to visualize cell nuclei. Nuclear morphological changes were then observed by fluorescence microscopy. Arrows indicate (1) chromatin lysis, (2) nuclear condensation, and (3) aggregation of apoptotic bodies.

To gain insight on how the HUE biosynthesized AuNPs inhibit cancer cell viability, we investigated the induction of apoptosis in AuNPs-treated MDA-MB-231 breast cancer cells. The morphological characteristics of cells were examined after 24 h of treatment with AuNPs using an inverted phase-contrast microscope. Analysis of the images showed a AuNPs concentration dependent decrease in the total number of cells per microscopic field. Additionally, apoptosis was confirmed by the presence of apoptotic bodies, echinoid spikes, and membrane blebbing (Fig. 9A). Further analysis of AuNPs-treated and DAPI-stained cells showed chromatin lysis, condensation of nuclear material, and aggregation of apoptotic bodies (Fig. 9B). Taken together, these results strongly indicate that the anticancer potential of HUE synthesized AuNPs is accompanied by the induction of apoptosis.

## Conclusion

Plant-mediated synthesis of AuNPs has received significant attention due to the accessibility, reproducibility, safety, and cost-effectiveness of plants. In addition, the abundance and diversity of plant species provide a rich source of phytochemicals for the synthesis of nanoparticles^44^. Moreover, the sustainable preparation of nanoparticles contributes significantly to the United Nations Sustainable Development Goals SDG 3 (Good Health and Well-Being), SDG 6 (Clean Water and Sanitation), SDG 9 (Industry, Innovation, and Infrastructure), and SDG 12 (Responsible Consumption and Production) by minimizing the use of hazardous chemicals, reducing the generation and release of waste in the environment, and promoting the use of industrial practices with low energy consumption^45^.

Generally, plant-mediated synthesis of nanoparticles is straightforward, with nanoparticles forming spontaneously when metal precursors are combined with plant material. Factors such as concentration, incubation time, pH, temperature, and solvents influence the morphology, size, and surface properties of the synthesized nanoparticles^46–48^. Biogenic AuNPs have been used in various fields, including agriculture, food industry, biomedicine, and textiles, due to their safety profile and enhanced properties. Biogenic AuNPs are also successfully used in drug delivery, diagnosis, and imaging^49^. Their nontoxicity, non-immunogenicity, and surface functionalization properties make them ideal candidates for targeted drug delivery.

In the present study, we report for the first time the use of the ethanolic extract of *H. uninervis* (HUE) for the successful synthesis of gold nanoparticles via a one-pot, green synthesis route. The biogenic AuNPs absorbed at λ_abs_ = 550 nm, confirming successful production of AuNPs^28^. Further characterization revealed the production of spherical AuNPs that are uniform and less than 50 nm in size. X-ray diffraction (XRD) indicated that the biogenic AuNPs are crystalline in nature, which is consistent with other biogenic AuNPs using different plant extracts^31–33^. Besides its role as a reducing agent, we demonstrated that HUE exhibits a protective role on the surface of AuNPs by acting as a stabilizing/capping agent. Fourier Transform Infrared Spectroscopy (FTIR) spectral measurements revealed the presence of hydroxyl and carbonyl functional group that are usually associated with different classes of phytochemical compounds^31,50–57^. This could explain the role of bioactive metabolites present in HUE in the reduction, capping, and stabilization of the biogenic AuNPs. The anticancer potential of biogenic AuNPs was revealed by their ability to inhibit cell viability and induce apoptosis in several cancer cell lines. These AuNPs showed a higher anticancer effect on cancer cells (using lower concentrations) compared to *H. uninervis* extract alone^58^, making it a promising approach to cancer treatment as it was shown to be more efficient and targeted.

While our results revealed potential anticancer activity of HUE synthesized AuNPs in vitro, our work has several limitations and challenges to be highlighted. It is important to translate our findings into an in vivo model for the evaluation of AuNPs safety, efficacy, and pharmacokinetics. In vivo studies, however, are associated with some limitation and challenges, including AuNPs biocompatibility, potential toxicity, targeted activity, as well as immune system activation. It is thus necessary to perform detailed mechanistic investigations of HUE synthesized AuNPs in vivo to have a better understanding of how these nanoparticles might perform in a clinical setting. Another challenge lies in scaling up the production of these biogenic AuNPs for medical, environmental, or even industrial applications^59^. Plant-based synthesis is often associated with low yield, and the process could be costly, especially when accounting for purification and quality control. The availability and sustainability of plant resources also contributes to the complexity of large-scale production. Therefore, it is crucial to optimize the synthesis process to ensure consistency and reproducibility of the size, shape, and concentration of the synthesized AuNPs as these characteristics significantly impact their effectiveness in various applications. The synthesis of AuNPs using *H. uninervis* poses potential environmental risks. For instance, harvesting large quantities of *H. uninervis* could disrupt local marine ecosystems, as these seagrasses are known for their critical roles in coastal environments. Therefore, comprehensive environmental impact assessments are obligatory when considering large-scale production of AuNPs form *H. uninervis*.

To the best of our knowledge, this work provides for the first time an insight on the use of HUE in the green synthesis of AuNPs with anticancer activity. The significance of our work lies in discovering a novel extract from a widely available seagrass that could potentially be used in the development of a therapeutic anticancer agent. Moreover, HUE provides a mean for the biosynthesis of AuNPs that are eco-friendly, cost-effective, biocompatible, nontoxic, and with enhanced activities. These AuNPs could offer an alternative therapeutic approach for targeting cancer and other chronic inflammation-mediated diseases. Additionally, these AuNPs could be further optimized by functionalizing their surface with targeting ligands or drugs to enhance their specificity and uptake by cells. AuNPs could also be combined with conventional chemotherapeutics to accomplish a synergistic anticancer effect. Overall, our work underscores the value of *Halodule uninervis* as a source for the green synthesis of AuNPs with pharmacological potential, particularly for the development of novel therapeutic agents with anticancer and/or anti-inflammatory activity.

## Data Availability

Data generated or analysed during this study are included in this published article. Some raw datasets used and/or analyzed during the current study and are not included are available from the corresponding author on reasonable request.

## References

[1] Yaqoob, A. A. et al. Recent advances in metal decorated nanomaterials and their various biological applications: a review. *Front. Chem.* **8**, 341 (2020). 32509720 10.3389/fchem.2020.00341 PMC7248377

[2] Kazmi, S. A. R., Qureshi, M. Z. & Masson, J-F. Drug-based gold nanoparticles overgrowth for enhanced SPR biosensing of doxycycline. *Biosensors* **10**(11), 184 (2020). 33228248 10.3390/bios10110184 PMC7699512

[3] Hainfeld, J. F., Smilowitz, H. M., O’connor, M. J., Dilmanian, F. A. & Slatkin, D. N. Gold nanoparticle imaging and radiotherapy of brain tumors in mice. *Nanomedicine* **8**(10), 1601–1609 (2013). 23265347 10.2217/nnm.12.165 PMC3657324

[4] Zhang, Y. et al. Temperature-dependent cell death patterns induced by functionalized gold nanoparticle photothermal therapy in melanoma cells. *Sci. Rep.* **8**(1), 8720 (2018). 29880902 10.1038/s41598-018-26978-1 PMC5992202

[5] Sathiyaraj, S. et al. Biosynthesis, characterization, and antibacterial activity of gold nanoparticles. *J. Infect. Public Health* **14**(12), 1842–1847 (2021). 34690096 10.1016/j.jiph.2021.10.007

[6] Brown, S. D. et al. Gold nanoparticles for the improved anticancer drug delivery of the active component of oxaliplatin. *J. Am. Chem. Soc.* **132**(13), 4678–4684 (2010). 20225865 10.1021/ja908117a PMC3662397

[7] Bai, X. et al. The basic properties of gold nanoparticles and their applications in tumor diagnosis and treatment. *Int. J. Mol. Sci.* **21**(7), 2480 (2020). 32260051 10.3390/ijms21072480 PMC7178173

[8] Hussain, M. H. et al. Synthesis of various size gold nanoparticles by chemical reduction method with different solvent polarity. *Nanoscale Res. Lett.* **15**, 1–10 (2020). 32617698 10.1186/s11671-020-03370-5 PMC7332595

[9] Jana, N. R., Gearheart, L. & Murphy, C. J. Evidence for seed-mediated nucleation in the chemical reduction of gold salts to gold nanoparticles. *Chem. Mater.* **13**(7), 2313–2322 (2001).

[10] Fazio, E. et al. Nanoparticles engineering by pulsed laser ablation in liquids: concepts and applications. *Nanomaterials* **10**(11), 2317 (2020). 33238455 10.3390/nano10112317 PMC7700616

[11] Amendola, V., Polizzi, S. & Meneghetti, M. Laser ablation synthesis of gold nanoparticles in organic solvents. *J. Phys. Chem. B* **110**(14), 7232–7237 (2006). 16599492 10.1021/jp0605092

[12] Ali, S. et al. State of the art of gold (au) nanoparticles synthesis via green routes and applications: a review. *Environ. Nanatechnol. Monit. Manag.* **16**, 100511 (2021).

[13] Muniyappan, N., Pandeeswaran, M. & Amalraj, A. Green synthesis of gold nanoparticles using Curcuma pseudomontana isolated curcumin: its characterization, antimicrobial, antioxidant and anti-inflammatory activities. *Environ. Chem. Ecotoxicol.* **3**, 117–124 (2021).

[14] Akhtar, S. et al. Formulation of gold nanoparticles with hibiscus and curcumin extracts induced anti-cancer activity. *Arab. J. Chem.* **15**(2), 103594 (2022).

[15] Chahardoli, A., Karimi, N., Sadeghi, F. & Fattahi, A. Green approach for synthesis of gold nanoparticles from Nigella Arvensis leaf extract and evaluation of their antibacterial, antioxidant, cytotoxicity and catalytic activities. *Artif. Cells Nanomed. Biotechnol.* **46**(3), 579–588 (2018). 28541741 10.1080/21691401.2017.1332634

[16] Padalia, H. & Chanda, S. Antioxidant and anticancer activities of gold nanoparticles synthesized using aqueous leaf extract of Ziziphus nummularia. *BioNanoScience* **11**, 281–294 (2021).

[17] Kim, D. H. et al. Nutritional and bioactive potential of seagrasses: a review. *South. Afr. J. Bot.* **137**, 216–227 (2021).

[18] de la Torre-Castro, M. & Rönnbäck, P. Links between humans and seagrasses—an example from tropical East Africa. *Ocean. Coastal. Manag.* **47**(7–8), 361–387 (2004).

[19] Baehaki, A., Lestari, S., Hendri, M. & Ariska, F. Antidiabetic activity with N-hexane, ethyl-acetate and ethanol extract of *Halodule uninervis* seagrass. *Pharmacogn. J.* **12**(4), 1 (2020).

[20] Karthikeyan, R. & Sundarapandian, M. Antidiabetic activity of methanolic extract of Halodule uninervis in Streptozotocin-induced diabetic mice. *J. Pharm. Sci. Res.* **9**(10), 1864–1868 (2017).

[21] Supriadi, A., Baehaki, A. & Pratama, M. C. Antibacterial activity of methanol extract from seagrass of Halodule uninervis in the coastal of Lampung. *Pharm. Lett.* **8**, 77–79 (2016).

[22] Ghandourah, M., Hawas, U. W., Abou El-Kassem, L. T. & Shaher, F. M. Fatty acids and other chemical compositions of some seagrasses collected from the Saudi Red Sea with potential of antioxidant and anticancer agents. *Thalassas Int. J. Mar. Sci.* **37**, 13–22 (2021).

[23] Ramah, S. et al. Prophylactic antioxidants and phenolics of seagrass and seaweed species: a seasonal variation study in a Southern Indian Ocean Island, Mauritius. *Internet J. Med. Update Ejournal* **9**(1), 27–37 (2014).

[24] Parthasarathi, P., Umamaheswari, A., Banupriya, R. & Elumalai, S. Phytochemical screening and in-vitro anticancer activity of ethyl acetate fraction of Seagrass *Halodule uninervis* from Mandapam Coastal Region Rameswaram Gulf of Mannar India. *Int. J. Pharm. Sci. Drug Res.* **13**(6), 677–684 (2021).

[25] Mayer, K. M. & Hafner, J. H. Localized surface plasmon resonance sensors. *Chem. Rev.* **111**(6), 3828–3857 (2011). 21648956 10.1021/cr100313v

[26] Hutter, E. & Fendler, J. H. Exploitation of localized surface plasmon resonance. *Adv. Mater.* **16**(19), 1685–1706 (2004).

[27] Petryayeva, E. & Krull, U. J. Localized surface plasmon resonance: nanostructures, bioassays and biosensing—A review. *Anal. Chim. Acta* **706**(1), 8–24 (2011). 21995909 10.1016/j.aca.2011.08.020

[28] Oliveira, A. E. F., Pereira, A. C., Resende, M. A. & Ferreira, L. F. Gold nanoparticles: a didactic step-by-step of the synthesis using the Turkevich method, mechanisms, and characterizations. *Analytica* **4**(2), 250–263 (2023).

[29] El-Brolossy, T. et al. Shape and size dependence of the surface plasmon resonance of gold nanoparticles studied by photoacoustic technique. *Eur. Phys. J. Spl. Top.* **153**, 361–364 (2008).

[30] Lee, K-S. & El-Sayed, M. A. Gold and silver nanoparticles in sensing and imaging: sensitivity of plasmon response to size, shape, and metal composition. *J. Phys. Chem. B* **110**(39), 19220–19225 (2006). 17004772 10.1021/jp062536y

[31] Islam, N. U., Amin, R., Shahid, M. & Amin, M. Gummy gold and silver nanoparticles of apricot (Prunus armeniaca) confer high stability and biological activity. *Arab. J. Chem.* **12**(8), 3977–3992 (2019).

[32] Patra, J. K., Kwon, Y. & Baek, K-H. Green biosynthesis of gold nanoparticles by onion peel extract: synthesis, characterization and biological activities. *Adv. Powder Technol.* **27**(5), 2204–2213 (2016).

[33] Ghodake, G., Deshpande, N., Lee, Y. & Jin, E. Pear fruit extract-assisted room-temperature biosynthesis of gold nanoplates. *Colloids Surf. B Biointerfaces* **75**(2), 584–589 (2010). 19879738 10.1016/j.colsurfb.2009.09.040

[34] Bjelajac, A. et al. Gold nanoparticles synthesis and immobilization by atmospheric pressure DBD plasma torch method. *Nanoscale Adv.* **5**(9), 2573–2582 (2023). 37143807 10.1039/d3na00007a PMC10153074

[35] Ogundare, O. D., Akinribide, O. J., Adetunji, A. R., Adeoye, M. O. & Olubambi, P. A. Crystallite size determination of thermally deposited gold nanoparticles. *Procedia Manuf.* **30**, 173–179 (2019).

[36] Dilipan, E., Sivaperumal, P., Kamala, K., Ramachandran, M. & Vivekanandhan, P. Green synthesis of silver nanoparticles using seagrass *Cymodocea serrulata* (R. Br.) Asch. & Magnus, characterization, and evaluation of anticancer, antioxidant, and antiglycemic index. * Biotechnol. Appl. Biochem.* (2023). 10.1002/bab.2444 36724497

[37] Rajeswaran, S., Somasundaram Thirugnanasambandan, S., Rengasamy Subramaniyan, S., Kandasamy, S. & Vilwanathan, R. Synthesis of eco-friendly facile nano-sized zinc oxide particles using aqueous extract of Cymodocea serrulata and its potential biological applications. *Appl. Phys. A* **125**, 1–12 (2019).

[38] B Chanthini, A. et al. Structural characterization, antioxidant and in vitro cytotoxic properties of seagrass, *Cymodocea serrulata* (R. Br.) Asch. & Magnus mediated silver nanoparticles. *J. Photochem. Photobiol. B Biol.* **153**, 145–152 (2015). 10.1016/j.jphotobiol.2015.09.014 26409094

[39] Nagaraja, S. K. et al. Biomimetic synthesis of silver nanoparticles using *Cucumis sativus* var. Hardwickii fruit extract and their characterizations, anticancer potential and apoptosis studies against Pa-1 (human ovarian teratocarcinoma) cell line via flow cytometry. *Appl. Nanosci.* **13**(4), 3073–3084 (2023).

[40] Math, H. H. et al. Investigation of in vitro anticancer and apoptotic potential of biofabricated silver nanoparticles from *Cardamine hirsuta* (L.) leaf extract against Caco-2 cell line. *Inorganics* **11**(8), 322 (2023).

[41] Shashiraj, K. N. et al. Exploring the antimicrobial, anticancer, and apoptosis inducing ability of biofabricated silver nanoparticles using *Lagerstroemia speciosa* flower buds against the human osteosarcoma (MG-63) cell line via flow cytometry. *Bioengineering* **10**(7), 821 (2023). 37508848 10.3390/bioengineering10070821 PMC10376666

[42] Shashiraj, K. N. et al. Rotheca Serrata flower bud extract mediated bio-friendly preparation of silver nanoparticles: their characterizations, anticancer, and apoptosis inducing ability against pancreatic ductal adenocarcinoma cell line. *Processes* **11**(3), 893 (2023).

[43] Nagaraja, S. K., Niazi, S. K., Bepari, A., Assiri, R. A. & Nayaka, S. Leonotis nepetifolia flower bud extract mediated green synthesis of silver nanoparticles, their characterization, and in vitro evaluation of biological applications. *Materials* **15**(24), 8990 (2022). 36556796 10.3390/ma15248990 PMC9781718

[44] Osman, A. I. et al. Synthesis of green nanoparticles for energy, biomedical, environmental, agricultural, and food applications: a review. *Environ. Chem. Lett.* **22**(2), 841–887 (2024).

[45] Nyandoro, V. O., Masioge, H. K. & Malago, Z. L. Biogenic synthesis of metal nanoparticles: promoting green nanotechnology and sustainable development goals. *Clean Technol. Environ. Policy* 1–14 (2024).

[46] Satpathy, S., Patra, A., Ahirwar, B. & Hussain, M. D. Process optimization for green synthesis of gold nanoparticles mediated by extract of *Hygrophila spinosa* T. Anders and their biological applications. *Phys. E Low-dimens. Syst. Nanostruct.* **121**, 113830 (2020).

[47] Casagrande, M. et al. Influence of time, temperature and solvent on the extraction of bioactive compounds of Baccharis dracunculifolia: in vitro antioxidant activity, antimicrobial potential, and phenolic compound quantification. *Ind. Crops Prod.* **125**, 207–219 (2018).

[48] Yasmin, A., Ramesh, K. & Rajeshkumar, S. Optimization and stabilization of gold nanoparticles by using herbal plant extract with microwave heating. *Nano Converg.* **1**(1), 12 (2014). 28191395 10.1186/s40580-014-0012-8 PMC5270968

[49] Kong, F-Y. et al. Unique roles of gold nanoparticles in drug delivery, targeting and imaging applications. *Molecules* **22**(9), 1445 (2017). 28858253 10.3390/molecules22091445 PMC6151763

[50] Botteon, C. et al. Biosynthesis and characterization of gold nanoparticles using Brazilian red propolis and evaluation of its antimicrobial and anticancer activities. *Sci. Rep.* **11**(1), 1974 (2021). 33479338 10.1038/s41598-021-81281-w PMC7820602

[51] Rodríguez-León, E. et al. Synthesis of gold nanoparticles using *Mimosa tenuiflora* extract, assessments of cytotoxicity, cellular uptake, and catalysis. *Nanoscale Res. Lett.* **14**, 1–16 (2019). 31654146 10.1186/s11671-019-3158-9 PMC6814701

[52] Park, S. Y., Yi, E. H., Kim, Y. & Park, G. Anti-neuroinflammatory effects of Ephedra sinica Stapf extract-capped gold nanoparticles in microglia. *Int. J. Nanomed.* **1**, 2861–2877 (2019). 10.2147/IJN.S195218 PMC6497913 31118612

[53] Benedec, D. et al. *Origanum vulgare* mediated green synthesis of biocompatible gold nanoparticles simultaneously possessing plasmonic, antioxidant and antimicrobial properties. *Int. J. Nanomed.* 1041–1058 (2018). 10.2147/IJN.S149819 PMC5824763 29503540

[54] Elbagory, A. M., Meyer, M., Cupido, C. N. & Hussein, A. A. Inhibition of bacteria associated with wound infection by biocompatible green synthesized gold nanoparticles from South African plant extracts. *Nanomaterials* **7**(12), 417 (2017). 29186826 10.3390/nano7120417 PMC5746907

[55] Zhang, P., Wang, P., Yan, L. & Liu, L. Synthesis of gold nanoparticles with *Solanum xanthocarpum* extract and their in vitro anticancer potential on nasopharyngeal carcinoma cells. *Int. J. Nanomed.* 7047–7059 (2018). 10.2147/IJN.S180138 PMC6219121 30464458

[56] Ismail, E. H., Saqer, A. M., Assirey, E., Naqvi, A. & Okasha, R. M. Successful green synthesis of gold nanoparticles using a *Corchorus olitorius* extract and their antiproliferative effect in cancer cells. *Int. J. Mol. Sci.* **19**(9), 2612 (2018). 30177647 10.3390/ijms19092612 PMC6163711

[57] Alexeree, S. M., Sliem, M. A., El-Balshy, R. M., Amin, R. M. & Harith, M. Exploiting biosynthetic gold nanoparticles for improving the aqueous solubility of metal-free phthalocyanine as biocompatible PDT agent. *Mater. Sci. Eng. C* **76**, 727–734 (2017). 10.1016/j.msec.2017.03.129 28482583

[58] Wehbe, N. et al. The antioxidant potential and anticancer activity of *Halodule uninervis* ethanolic extract against triple-negative breast cancer cells. *Antioxidants* **13**(6), 726 (2024). 38929164 10.3390/antiox13060726 PMC11200955

[59] Bharali, A., Deka, B., Sahu, B. P. & Laloo, D. Major challenges and probable scientific solutions toward the large-scale production of plant-based metallic nanoparticles: a systematic review. *Nanatechnol. Environ. Eng.* **8**(4), 933–941 (2023).

